# Editorial: Hardware implementation of spike-based neuromorphic computing and its design methodologies

**DOI:** 10.3389/fnins.2022.1113983

**Published:** 2023-01-06

**Authors:** Lining Zhang, Mansun Chan

**Affiliations:** ^1^School of Electronic and Computer Engineering, Peking University, Shenzhen, China; ^2^Department of Electronic and Computer Engineering, Hong Kong University of Science and Technology, Hong Kong, Hong Kong SAR, China; ^3^ACCESS-AI Chip Center for Emerging Smart Systems, InnoHK Centers, HKSP, Hong Kong, Hong Kong SAR, China

**Keywords:** spiking neural network, hardware SNN, neuromorphic computing, SNN co-designs, design methodology

Spiking Neural Network (SNN), as the third-generation neural network, mimics the operations of brains and integrates the memory (synapse) and processing units (neurons) in proximity. Implementing the SNN in a modular, parallel, distributed and scalable manner, promises a computing system with low power consumption and short latency (Berggren et al., 2021; Christensen et al., 2022) The biologically plausible, hardware based neuromorphic computing, based on mature CMOS technologies or emerging memristor technologies (Zhu et al., 2020), is revolutionary and excels in the computations of spatial-temporal dynamics with plasticity and fault-tolerance.

This Research Topic achieves five excellent papers related to design and deployment of SNNs on hardware, including design methodologies, system implementations, and benchmarking against those with von Neumann architecture. For implementations into available SNN frameworks, algorithm-hardware co-design is generally the approach to follow. Either starting directly from an SNN design or starting with an artificial neural network (ANN) then converting to an SNN, simulations with the neural network module taking into account hardware properties, is the first step toward hardware implementation. It is similar to the use of electronic design automations (EDA) tools for the CMOS integrated circuit designs. Similar design methodology has already been used in memristor-based neuromorphic computing systems (Ishii et al., 2019), but remains in small scale. Looking into the future, neuromorphic computing-oriented EDA (may be coined as NC-EDA) is going to grow together with the hardware in the era of non-von Neumann computing.

In their work, Patiño-Saucedo et al. developed a method to implement liquid state machines (LSMs) into SpiNNaker to classify visual event. They design their LSMs with an input layer, a hidden layer with recurrently connected SNNs and an output layer, on top of the PyTorch module. The training of hidden-to-output parameters is done using the spatio temporal back propagation algorithm by keeping the other weights untrained. LSMs parameters are extracted and then used in an equivalent LSM model defined with a PyNN-based API of the SpiNNaker. After setting the parameters using the specific software of the SpiNNaker platform, the authors map them into the hardware and perform inference on the N-MNIST dataset. Benchmarks show that an accuracy of 93.9% is achieved with a liquid size of 4,096. The effect of weight quantization is also discussed from the perspective of accuracy.

A second hardware implementation of neuromorphic computing was proposed by LeBow et al. The application scenario is a real-time edge neuromorphic tasting system. In order to deploy the SNN into the Intel Loihi neuromorphic chip in the Kapoho Bay USB stick form factor, the authors trained a convolutional neural network (CNN) with a kernel size of 4 and 32 convolutional kernels per layer. The training is performed in Keras and then converted to a rate-based SNN using the SNN Toolbox. The NxTF framework with a Keras-like API allows the deployment of the converted SNN on Loihi. Sensors, dataset, etc. have also been discussed in the work thoughtfully and made available to the community. Significant advantages over other devices are achieved. The 15 mW dynamic power is 49 and 643 times lower than the same model on non-spiking ANN and GPU, respectively, with similar accuracy. In terms of inference energy, the SNN is 15 and 290 times lower, showing very promising advantages.

Li et al. discussed their proposal of quantization framework for fast spiking neural networks. While the ANN-to-SNN conversion is one way to develop neuromorphic hardware, traditional conversion suffers a trade-off between the accuracy and latency. It is difficult to maintain accuracy with low activation bit-width. The authors propose to perform learned step size quantization (LSQ) first before the ANN-to-SNN conversion. Furthermore, the authors develop ways to reduce occasional noise and model the max pooling for SNN, and eventually derive the lossless quant-ANN-to-SNN conversion. Using the proposed technique, the authors achieve a large increase in accuracy with limited latency, i.e., 70.18% accuracy on ImageNet within eight time-steps. The work pioneers fast ANN-to-SNN conversions.

Gao et al. presented their work on SNN implementation in a field-programmable gate array (FPGA). Instead of converting ANNs to SNNs, the authors started directly from an SNN architecture design. The SNN consists of three layers, while the hidden layer consists of three compartments, i.e., a multi-compartment leaky integrated-and-fire (MLIF) model. Targeting for online training, feedback from the output layer to the apical dendrite is introduced for error backpropagations. The output layer also consists of two compartments and a teaching current is added to the soma neurons. A training is designed that consists of two stages, the forward stage and the target stage. At the end of the target stage, the synaptic weights and biases in the hidden layer are updated according to the plateau potential of the dendrites of the output layer. The authors simulated their SNN design on MATLAB before deploying into the Alterra FPGA. Benchmarks of FPGA implementations including R-square metrics are reported with encouraging results.

A review of neuromorphic computing systems is presented by Ivanov et al. From their perspective, the authors proposed a list of neuromorphic properties to characterize neuromorphic computing hardware, including connectivity, parallelism, asynchrony, the impulse nature of information transfer, online learning, local learning, sparsity, analog computing, and in-memory computing. Looking around, authors introduce several popular neuromorphic projects from industry and academia, including TrueNorth, Loihi, Tianjic, SpiNNaker, BrainScales, NeuroFlow, DYNAP, Akida, and Mythic. A comparison between different projects is also performed from several ways.

## Author contributions

LZ and MC drafted and reviewed the manuscript. All authors contributed to the article and approved the submitted version.
